# Perceptions of cannabis as a stigmatized medicine: a qualitative descriptive study

**DOI:** 10.1186/1477-7517-10-2

**Published:** 2013-02-16

**Authors:** Joan L Bottorff, Laura JL Bissell, Lynda G Balneaves, John L Oliffe, N Rielle Capler, Jane Buxton

**Affiliations:** 1 School of Nursing, Faculty of Health and Social Development, University of British Columbia’s Okanagan campus, Kelowna, BC, V1V 1V7, Canada; 2 Faculty of Health and Social Development, University of British Columbia’s Okanagan campus, Kelowna, BC, V1V 1V7, Canada; 3 School of Nursing, University of British Columbia, T201 2211 Wesbrook Mall, Vancouver, BC, V6T 2B5, Canada; 4 Interdisciplinary Graduate Studies Program, University of British Columbia, T201 2211 Wesbrook Mall, Vancouver, BC, V6T 2B5, Canada; 5 School of Population and Public Health, University of British Columbia, 171 - 2206 East Mall, Vancouver, V6T 1Z3, Canada; 6 Institute for Healthy Living and Chronic Disease Prevention, University of British Columbia’s Okanagan campus, 3333 University Way, Kelowna, BC, V1 1V7, Canada

**Keywords:** Cannabis, Medical marijuana, Stigma, Cannabis, Legal consequences, Social consequences

## Abstract

**Background:**

Despite its increasing prevalence and acceptance among the general public, cannabis use continues to be viewed as an aberrant activity in many contexts. However, little is known about how stigma associated with cannabis use affects individuals who use cannabis for therapeutic purposes (CTP) and what strategies these individuals employ to manage associated stigma. The aim of this Canadian study was to describe users’ perceptions of and responses to the stigma attached to using CTP.

**Methods:**

Twenty-three individuals who were using CTP for a range of health problems took part in semi-structured interviews. Transcribed data were analyzed using an inductive approach and comparative strategies to explore participants’ perceptions of CTP and identify themes.

**Results:**

Participant experiences of stigma were related to negative views of cannabis as a recreational drug, the current criminal sanctions associated with cannabis use, and using cannabis in the context of stigmatizing vulnerability (related to existing illness and disability). Strategies for managing the resulting stigma of using CTP included: keeping CTP ‘undercover’; educating those who did not approve of or understand CTP use; and using cannabis responsibly.

**Conclusions:**

Understanding how individuals perceive and respond to stigma can inform the development of strategies aimed at reducing stigma associated with the use of CTP and thereby address barriers faced by those using this medicine.

## 

Concurrent with its increasing use as an illegal recreational drug, a growing number of studies have highlighted the medical benefits of cannabis for diverse health conditions [1,2]. In 2001, the Canadian government officially created a medical cannabis programme to authorize the possession, production and distribution of cannabis for therapeutic purposes (CTP) for individuals meeting specific criteria. Nevertheless, researchers report that cannabis use continues to be viewed as aberrant and CTP users experience stigma related to their use of cannabis [3]. The goal of this study was to describe users’ perceptions of and responses to the stigma they experience related to CTP in order to provide a foundation for developing strategies for reducing the stigma and supporting CTP users in their use of this medicine.

## Background

Notwithstanding its current illegal status in Canada, cannabis has become the most widely used illicit drug and its use is on the rise among most population groups [4]. In British Columbia, Canada, the setting of the current research, over 50% of the population 15 years and older have consumed cannabis at least once in their lives [5]. As a result, consuming cannabis has transitioned from a once underground activity to one more openly accepted by many. Public opinion continues to shift towards the elimination or reduction of criminal penalties for cannabis-related activities. However, those who continue to believe these activities should be penalized are increasingly more likely to hold favourable attitudes toward cannabis when it is used for strictly therapeutic benefits [6,7]. Despite these changes in public attitudes towards cannabis, users continue to experience a certain level of stigma and risk in their use of CTP, particularly from authorities such as employers, landlords, and law enforcement [3,8]. Specific civic norms and etiquette are often employed by users in public spaces to avoid drawing attention to their cannabis use. Even with the establishment of Health Canada’s Canada Medical Marihuana Access Regulations (MMAR) in 2001 stigma against CTP users remains an issue [9]. Little is known about how the stigmatization of cannabis use influences therapeutic users’ patterns of use and their personal lives, and in-depth explorations of the strategies they employ to manage these experiences are limited.

### Stigma, health and CTP

Goffman’s (1963) ground breaking work on stigma underpins our understanding of health-related stigma [10]. In this work, he defines stigma as: “*The phenomenon whereby an individual with an attribute which is deeply discredited by his/her society is rejected as a result of the attribute”* (p.21) and argues that stigma is an interactional process that “spoils identity.” As such, people who are perceived by others to deviate physically and behaviourally from social norms and values are subject to disapproval, and marginalization, and often experience discrimination and loss of status. These social interactions can result in enacted stigma (i.e., external stigma) when others’ judgements about difference are translated into rejection, distancing and other discriminatory practices; as well as perceived stigma (i.e., internal stigma) wherein individuals’ assumptions or fears of discrimination lead to self-perceptions of shame and guilt, and protective action such as self-imposed isolation.

Efforts to use and refine the concept of stigma for public health have been prompted by observations of the profound negative health effects of the social disqualification of individuals and groups who are identified with particular diseases or disorders [11,12]. For example, disease-related stigma in the context of mental health problems highlight the significant deleterious effect of stigma on health and well-being when individuals avoid health care or seeking other forms of support because of feelings of shame or embarrassment.

As chronically ill individuals and illicit drug users, CTP users are at a high risk of experiencing multiple sources of stigma from various fronts [13]. A diagnosis of chronic illness that is either visible (e.g., multiple sclerosis, and epilepsy) or relatively invisible (e.g., HIV/AIDS, fibromyalgia, and mental illness) often results in stigma and social isolation [14-17]. Although substance use is associated with varying degrees of stigma, illicit drug users are among the most stigmatized groups [18,19]. Beyond the stigma of being labelled a drug user, the additional stigma of being formally charged as a criminal can also have lasting negative effects. Social disqualifications targeting other features of a person's identity (e.g., poverty, gender, sexual orientation) can compound these experiences of stigma [20,21]. An understanding of the experiences of stigma among CTP users is, therefore, important and relevant to the health services provided to these individuals.

### Social and legal consequences of using CTP

While studies that investigate the experiences and concerns of recreational cannabis users are common, CTP remains poorly understood. A Canadian study of current HIV/AIDS CTP users reported many CTP users were met with “laughter, scepticism, or with negative reactions” (p. 41) from non-users for their CTP use [22]. They felt stigmatized for their choice of therapy both by their “healthy” peers and the medical system in general. Becoming licensed users through the MMAD Health Canada program helped alleviate some of the related stress and perceived stigma of CTP use and empowered them to improve their overall health. Other authors have reported that social and legal concerns motivated some individuals to conceal their CTP use and avoid disclosure beyond immediate family members [23]. When CTP users met with disapproval from family members, they reported it was often based on concern over the legal implications of CTP use and the potential of negative health effects and addiction.

The negative social implications of using CTP have also been observed elsewhere. A California-based study of pregnant women suffering from Hyperemesis Gravidarum (a highly debilitating pre-partum illness characterized by severe nausea, vomiting, malnutrition and weight loss) found that for participants, cannabis was their best option when traditional treatments were ineffective and, at times, traumatic [24]. Being pregnant and using cannabis, however, put participants at high risk for stigmatization. They were often belittled and declared deviant by their peers and the medical community for their decision to use CTP. Additionally, while these women were open and successful in using cannabis to treat extreme symptoms, they continued to experience strong feelings of anxiety, guilt and fear over CTP use. As Canadian CTP regulations are now over a decade old, it is timely that research be conducted examining the social context of CTP use and the influence of stigma on CTP users’ lives. The specific research questions guiding this study were: 1) What are CTP users’ experiences of stigma? and 2) What strategies do CTP users employ to negotiate their experiences of stigma? By understanding how individuals perceive the potential social implications of CTP use, new approaches can be developed to reduce the stigma associated with CTP and support individuals using CTP cope with stigma.

## Methods

This research employed a qualitative descriptive design [25] and drew on the tenets of naturalistic inquiry [26] – a method recognised as particularly useful when investigating vulnerable persons with health disparities [27]. Using qualitative methods, inductive analysis and purposive sampling, we developed an in-depth account of the experiences of CTP users.

### Study setting

This study was conducted in Canada, where the use of CTP is directly shaped by the federal laws governing what is considered to be a controlled substance. Cannabis production, distribution and possession remain illegal in Canada, with the exception of Health Canada’s licensing program for therapeutic users, the Medical Marihuana Access Program (MMAP). Since the MMAP’s formation in 2001, those persons wishing to legally possess and obtain CTP must apply for a license directly to Health Canada, which acts as the governing body that oversees the implementation of the Medical Marihuana Access Regulations (MMAR). Paradoxically, Health Canada continues to state that “marihuana [sic] is not an approved therapeutic product” [28]. Ostensibly mixed messages, such as this policy statement, along with public health strategies directed towards reducing cannabis use (e.g., the National Anti-drug Strategy), has complicated the context within which individuals use CTP. Although the establishment of the MMAP has been seen as a step forward by some groups [29-31], others have expressed reservations about the program [9,32,33] pointing to access issues, the complexity of the application forms and the length of time required to process applications [9]. Apprehension about the quality, potency, and lack of quality control and strain selection of MMAD-supplied cannabis also continues to be a source of controversy for many CTP users [9]. Concerns about access have resulted in a recent court decision in Canada that has found the MMAR to be “constitutionally invalid and of no force and effect” [34], forcing Health Canada to engage in a community consultation process to discuss potential changes to the regulations and programme.

The need for safe and informed access to cannabis has been central to the development of community-based dispensaries (i.e., compassion clubs) in Canada. The dispensaries provide illegal, high quality cannabis to their members (who must have medical documentation of an approved medical condition) as well as information regarding CTP to assist with making decisions about cannabis use. The dispensaries reduce the risk of legal repercussions associated with accessing illegal cannabis by providing a safe environment for members to purchase CTP and by acting as members’ social and legal advocates [35]. Although access to CTP through dispensaries is a form of civil disobedience, many law enforcement officers and courts recognize identification cards from these dispensaries as adequate proof of legitimate CTP use, giving discharges to verified members and to dispensary operators who manage their clubs in a transparent and responsible manner [35].

### Recruitment and sampling

Following university ethical approval, purposive sampling was used to recruit current CTP users through four British Columbia community-based cannabis dispensaries as well as through a Canadian online forum of CTP users in 2007–2008. Eligibility criteria required participants to: a) report using CTP in the last 30 days and for over 6 consecutive months, b) be at least 19 years of age, and c) speak English. In accordance with ethical requirements and to protect individual identities, all participants reviewed the consent form and were asked to give their consent verbally on tape at the start of the interview. No record of participants’ names or identifying characteristics was kept and all participants received a C$25 honorarium for their time.

The sample comprised 23 participants (13 women, 10 men). Two transgendered (male to female) participants were included in the women’s subgroup. Participants ranged in age from 25 to 66 years (mean = 45 years) and had an average annual income of $21,000, slightly below Canada’s 2008 low income cut-off for individuals living in a large urban area ([36], p.25). Approximately 78% were either single or divorced/separated and over two thirds had completed at least some university or college. Most participants were engaged in paid work (52%) or caring for a family member (39%). HIV/AIDS was the most commonly reported disorder for which CTP was used (6 participants), followed by fibromyalgia (n = 5), arthritis (n = 4), mood/anxiety disorders (n = 3), cancer (n = 2), neurological disorders (n = 2), gender dysphoria (n = 2) and other disorders (n = 4). Some individuals were living with multiple diagnoses. Participants described their CTP use as long-term (mean = 8.3 years, range = 2 to 16 years). All participants smoked CTP; 15 indicated they also ingested it and nine used a vaporizer. Other methods used by participants included tinctures (n = 5), sprays (n = 2), cannabis mixed with tobacco (n = 1) and use of a poultice (cannabis mixed with alcohol and applied topically) (n = 1). When asked about their purchase locations, only five of the eleven participants that currently held a Health Canada license indicated they purchased their cannabis from Health Canada and most (n = 20) purchased it from a community-based dispensary. Participants also indicated they accessed CTP directly through licensed growers (n = 10) and non-licensed growers (n = 10).

### Data collection

Data were collected using semi-structured, individual face-to-face or telephone interviews. Participants were invited to discuss their beliefs about and experiences of using CTP and their experiences of stigma. At a time and location convenient to the participant, interviews were conducted by trained research assistants and lasted approximately 1–3 hours. A short questionnaire was administered to gather demographic data, history of cannabis use, and information about health issues influencing use of CTP.

### Data analysis

Using an inductive thematic approach to data analysis, interview transcripts were read and re-read by the authors and sections of the data that reflected emergent ideas and themes were highlighted. In investigative team meetings, independent reviews of the data were summated and shared to reach consensus about categories for coding the data. The qualitative data management software program, NVivo™, was used to organize the data for retrieval and in-depth analysis. Comparative strategies were used to explore participants’ perceptions of CTP.

## Results

Participants’ narratives included a predominant discourse of stigma associated with CTP use. Experiences of stigma arose in interactions with family members and close friends, as well as from others in society. The multiple dimensions of stigma associated with using CTP use identified in the data afforded a view of participants’ experiences whereby most contributed to more than one dimension. In order to achieve the benefits of cannabis use, participants had to negotiate social censorship, disapproval, threats, and isolation. Ways participants coped with and minimized their experiences of stigma associated with CTP use are also described.

### Dimensions of stigma associated with CTP

Three dimensions of stigma were identified that related to negative views of cannabis as a recreational drug, illegal activity surrounding cannabis use, and layered vulnerabilities related to poverty and particular illnesses and disabilities. Each dimension is described in the following sections.

#### Medicine in a joint

Unlike other medications the participants used, CTP was more difficult to conceal particularly when consumed through smoking. The distinctive and often times strong smell, appearance, and behaviours associated with smoking a joint invoke negative images for some, such as the “pothead,” and have been reinforced by the media and public opinion. We use the word “joint” deliberately to highlight the stigma participants’ experienced. Dominant views of cannabis, as a recreational drug used for pleasure, to just “get high” and to escape the realities of life were perceived to make it difficult for the medicinal value of cannabis to be recognized and defended in an objective way. As a consequence, participants reported being labelled as “potheads” by their families, healthcare providers and society at large. Some were falsely accused of using CTP not for medicinal purposes but “just to have some fun” (woman, aged 45, digestive disorder). These labels positioned CTP users as irresponsible, non-contributing, and on the margins of society, unbecoming attributions participants refuted. One man (aged 45, fibromyalgia) resented “being perceived as something less than acceptable” and felt that he was unfairly judged by others specifically because of his use of CTP:

"Nobody turns around and says you’re a junkie if you have terminal cancer and are on heroin. But it doesn’t matter why you’re on marijuana, [if] you’re on marijuana, “You’re a pothead and get the hell away from me.”"

In this example, the man reveals a comparison point whereby harder drugs such as heroin can be packaged as therapeutic and legitimate in the context of buffering the symptoms that accompany advanced disease when there is little hope of survival. Yet, cannabis is not understood as affording the same relief – rather, its use brings into question both the legitimacy of the illness and the role of smoked cannabis as a medicine. Constructions of cannabis as an addictive substance were also perceived to contribute to condemnations of its use as a medicinal drug of choice, and thereby stigmatized users. Users of CTP reported being labelled “drug addicts” and that others, including physicians, continually reminded users that cannabis was a “bad medicine” that could lead to addiction. Even when participants were prescribed other potentially addictive medications (e.g., oxycotin, sleeping pills), it was their use of cannabis that was scrutinized and criticized. Healthcare providers went as far as to offer participants counselling to “get help” with their assumed marijuana addiction.

External stigma was also reflected in the lack of trust expressed by family members as well as health professionals as a result of participants’ use of CTP. Participants reported not being believed by others when they described the medical benefits they experienced from cannabis and their requests for cannabis led to a questioning of the severity of their reported symptoms. Participants recounted that others thought they were “making things up,” “faking things” or “manipulating symptoms” to get safe access to cannabis. There was an underlying sense that participants were viewed as being unreliable, dangerous, unsavoury, and “abusing the system” when in fact, they believed they were attempting to resolve the health problems they experienced in a responsible way.

Perceptions that cannabis use “changed” people and interfered with their ability to think clearly and act responsibly also contributed to the stigmatization that CTP users experienced. Participants reported to be reluctant to tell their employers or coworkers of their CTP use, fearing that they would lose their professional status, and they and their work performance would be negatively judged.

In summary, there was consensus that the stigma associated with cannabis use negatively impacted participants’ social, professional and family ties as well as their relationships with healthcare providers. These reactions forced participants to self-regulate and withdraw from some of their social networks and resulted in social isolation, estrangement from family and friends, and for some, relocation to another city. The reactions also acted as a barrier to receiving the health care many participants needed.

#### Medicine on the wrong side of the law

Cannabis as a stigmatized medicine was also confounded with the fact that it is an illegal substance. Users of CTP, therefore, explained they were faced with not only being labelled as “potheads” but also criminals. They reported being viewed with suspicion and marginalized for their illegal activities associated with using CTP. One woman (aged 45, digestive disorder) indicated that she was initially hesitant to begin using CTP because of the stigma associated with cannabis as an illegal substance:

"When I first came to the compassion club it was an emotional thing for me, I cried when l left. I was like, “Oh my God, this is where my life has thrown me? I’ve lost my career. I’m in the ditch vomiting. Now this is what I have come to”. I was like, “It’s illegal! It’s illegal!” I want to be an upstanding citizen; I don’t want to be a criminal. But then, as I was realizing a little clearer what was really going on, I realized it was the biggest gift and my complete ally and then my whole concept just shifted."

Having a federal license or community-based dispensary membership card provided recognition of their need for medical cannabis and thus distinguished users of CTP from illegal recreational users. However, for some holding a license or membership card did not negate the stigma they experienced as CTP users because they felt “branded” as being involved in an apparently illegal activity, and described additional scrutiny and differential treatment that negatively impacted their lives. For example, a 55-year-old woman thought her fears would be relieved upon receiving her license from Health Canada, but instead felt much regret over the process and believed she was in a worse situation:

"I thought I’d feel different but I don’t… I don’t feel as safe now because I’ve identified myself as a pot smoker where before I was anonymous and I think I was in a better position… If I had to do it over again I wouldn’t even tell my doctor, it wasn’t worth it."

Similarly, a 27-year-old man with cancer believed that since receiving his Health Canada licence, he was “discriminated upon constantly” by police who would often detain him until they verified the legitimacy of his license:

"It’s all fun and games the first 10 times you do it but after, you know, you get pretty annoyed. I mean if I just had to flip them a card and walk away then that would be a little different but they’ve got to run your name. They’ve never heard of the program, they want to have it explained to them or if they have heard [of the MMAD], you know, I’ve literally had cops make me wait while they bring a couple of other cops over to look at the licence."

The inclination that those producing their own CTP might be dealers was also a site for stigma. Despite being “legal,” those that cultivated their own cannabis with licences were often harassed by local police, landlords and subsidised housing investigators. Several had been subjected to what they believed were unwarranted raids on their property and would often lose their cannabis plants in the process either due to confiscation by the police or by their own hand to conceal their gardens. One 36-year-old woman living with AIDS was repeatedly harassed by the police who were supposed to be checking the security of her residence. They wanted to see her garden and questioned the validity of her federal licence. Legal producers also had difficulty finding and keeping their housing due to landlords’ concerns about the legitimacy and impact on other tenants of their cultivation of cannabis. One participant, a man living with AIDS in a subsidised housing residence, complained that he was constantly investigated by the housing officials. He often dismantled his garden to avoid confrontation and to keep his lease despite the loss of his home-grown medicine.

Because of the current criminal sanctions associated with cannabis, participants believed their CTP use also raised suspicions and judgements about their ability to parent. Several participants feared losing custody of, or access to, their children as a result of being caught with CTP. One user of CTP (aged 34, AIDS) resented this, stating people “shouldn’t have to fear [their] kid being taken away because of [their] choice in medicine.” Being a parent, therefore, led participants to take steps to conceal their use of CTP.

#### Using cannabis in the context of layered vulnerabilities

For many participants, the stigmatization they experienced in using cannabis was entangled with other stigmatized vulnerabilities, such as living with a marginalized disorder (e.g., HIV/AIDS, fibromyalgia, mental illness, history of drug addiction), transitioning gender identity, being homosexual, or living in poverty. A 34-year-old man who held a federal licence, talked about the multiple stigmas he lived with which made his cannabis use less acceptable than that of others who did not have AIDS or a history of drug addiction:

"It doesn’t matter how many federal licences [I have]… I’ve got the stigma of AIDS, I’ve got the stigma of an ex-junkie, okay, so I’ve got a lot of dirt in my closet that can be thrown up, right. But if one of [my brother’s] friends who don’t have this dirt, if one of those friends suddenly started smoking cannabis and he got a federal licence like me, I think it would be a little more accepted."

In this example, the man’s history of addiction prevails and the remnants of his past drug use (i.e., HIV/AIDS) locate CTP as little more than a new addiction. These vulnerabilities created challenges in accessing CTP. Requests for CTP were often questioned or not taken seriously on the basis of already suspect diagnosis and practices, and frequently resulted in long delays in accessing CTP. Other individuals who had struggled for years to get diagnosed or be referred to specialists had difficulty generating enough energy to lobby or negotiate access to CTP when healthcare providers had already labelled them “problem” patients or held judgemental attitudes about their illnesses.

### Coping with stigma associated with CTP use

Choosing to continue their use of CTP because of the significant benefits experienced in relation to managing their health problems, participants engaged in a variety of coping strategies to respond to the stigma associated with CTP use. Strategies identified in this data were: keeping use of CTP undercover, convincing others of the benefits of CTP, being responsible in their use of CTP and actively defending their right to choose their own medication.

#### Covert use: keeping CTP use undercover

Some participants believed that with the overwhelming condemnation attributed to cannabis and the current criminal sanctions associated with cannabis in Canada, there was little they could do except be covert in their CTP use. As such, they guarded and hid their use of cannabis from others. When one 55-year-old woman was asked if she had any advice for other CTP users, she stated: “Keep your mouth shut, grow it, use it, don’t tell anybody, don’t even tell your family, don’t tell your friends, keep it to yourself and save your own life.” Individuals went to great length to cover up their CTP use, including lighting incense to mask the smell, smoking away from their home, changing their clothes after smoking cannabis, and being vigilant about who was around when they smoked.

By using CTP covertly, participants also protected themselves through self-imposed social isolation. Some isolated themselves in order to avoid criticism and feeling “guilty” about their use. Others smoked in private to avoid children seeing them smoke cannabis. One woman who isolated herself from her family explained:

"I have a very difficult time convincing my family why I have to use it and it’s just got to the point where I don’t even bother talking to my family because of the fact that they just keep dissing me because I use it.. They’re old school, a drug’s a drug, that’s their mentality."

#### Expert use: convincing others of the benefits of CTP use

Several participants believed that the harsh judgemental attitudes they had experienced were the result of “misinformation” from the media and a general lack of knowledge of CTP. As such, several participants believed that the only way to address this was to educate and discuss the therapeutic properties of cannabis “to open other people’s eyes.” One man (aged 42, daily user, AIDS) argued that if the perception of cannabis was to change to being a therapeutic agent rather than a recreational drug, much would be improved:

"It’s that stigma attached to pot, that lovely word pot has such a bad condemnation to it. Meanwhile people can pop sleeping pills left, right and center and nobody thinks anything of it. So it’s a perception. When we can change that perception of what this is and what the approach is [cannabis as therapy], the battle is half won. [It would help for] people to talk about the issue, get proper information out there, and if you can stack the seats with informed people and reach out to a community where you need to reach out to, then you can start the process."

The work of informing friends and family was often a long (but important) process of education on the part of participants. A 36-year-old woman’s experience with her mother typified this experience:

"She [participant’s mother] goes, “I think you have a problem, I think you have an addiction.” Now I looked at her and said “I’m not taking really any pain killers at all, okay, nothing, I’ve taken myself off prednisone, taken myself off the [mesalamine], not taking [acetaminophen/codeine], and you’re telling me, Mother, I’m possibly addicted to cannabis?” We had a slight fight about it [laughing] and then, of course, she changed her mind because I had to educate her, as well as many others, and now she doesn’t like to admit to that little story because now she is a full on cannabis granny, raging granny. I mean she is so supportive. Now she looks at me and she is very, very proud. She doesn’t feel I have an addiction problem in any way."

#### Responsible use: doing everything “right”

In an effort to reinforce the differences between recreational and therapeutic uses of cannabis, some participants cast aspersions on recreational users while exulting themselves as being a responsible user and “clean on other fronts” (aged 43, daily user, Fibromyalgia). For example, when asked how her therapeutic use compared to recreational users, one woman (aged 36, licensed user, HIV-AIDS) asserted, “They act stupid some of them…because they flaunt it, they’ll smoke it anywhere.” In contrast and as a “responsible” CTP user, she took precautions and always smoked with discretion: “I don’t flaunt it, like sit there with my arm out the window.” She identified recreational users as “pimps, pushers and, people in the criminal world” and stated they were “different” from her. A 36-year-old man (daily user, chronic back pain) believed therapeutic use was fundamentally different because “recreational people are the people who use it and giggle and laugh and joke around and then that’s it.” Participants perceived their use of CTP as “necessary” while recreational use was often strictly “social” in nature. A third participant (aged 36, daily user, HIV/AIDS) who indicated she never used cannabis recreationally stated: “I think the recreational is more for relaxation not for pain, what it’s supposed to be for, it’s more for them to party with. For us, it’s more of a life thing.” As a result of the necessity of their use of CTP, participants were very particular in how they procured their cannabis, how much they used, and when so as not to be confused with recreational drug addicts.

Leading by example was what one participant (aged 42, daily user, HIV/AIDS) believed he could do to change society’s perceptions of him and his CTP use. And while he was fully aware that he would not be able to change opinions overnight, he remained hopeful and believed that once others saw him as a responsible user, their attitudes towards him and CTP would start to change:

"I can only do what I can do for myself and present myself and approach my life in the way that shows that I am not a drug addict. I am not a detriment to society. I’m actually trying to be a part of society but I am kind of running into a lot of roadblocks. I know how the world works. It happens slowly, very slowly and usually it’s one or two or three people who start and take it somewhere and then other people build on it. That’s all you can do."

Participants also attempted to control the stigma surrounding their use of CTP by being open and honest about their use. Applying for a federally-issued licence for CTP use and production, and notifying law enforcement of their CTP production were ways some participants attempted to manage their image as a responsible cannabis user.

#### Activist use: CTP as a human rights issue

Notwithstanding the stigma experienced for using an illegal substance therapeutically, participants continued to staunchly defend their right to choose their own medication. And despite “swimming [in a] pool with sharks” and illegally accessing CTP, many participants were committed to using CTP and helping others gain access regardless of the potential risks, including arrest and/or imprisonment. Several participants became activists in their own right and argued that neither the government nor the medical community had the right to deny them access to their “medication”, or to persecute them for using it. Doing what he felt was “logically and ethically correct in [his] heart”, one 34-year-old man living with AIDS dared the government to take away his CTP:

"Screw them, I’m a free man, you know? Furthermore, I’m [now] like a 60 or 70 year old man. I’m living out my final years. Do you really think I’m going to listen to some federal regulation for Christ’s sake? I mean this is insane."

Similarly, other participants believed it was the duty and “moral ethical obligation” of Health Canada to explore the therapeutic uses of cannabis and to “open up access in order to maximize the benefits of medical cannabis in society as a whole”. Some were hopeful that through their activism, the laws surrounding CTP would eventually change and they would be able to use their medication freely and openly without fear of prosecution (woman aged 36, daily user, AIDS):

"I will get the message across, because I know it’s coming. Yeah, freedom is a right. I hope this all goes through finally [and] that we shouldn’t have to go to jail for what we believe in, for helping sick people. I don’t believe it’s a crime and I believe it’s a waste of taxpayer’s money, and the government should stay out of it. This should be a medical, a medical thing and that’s it."

## Discussion

Stigmatization as a form of social control which functions to discourage and penalize deviant behaviour, characteristics or identities was reflected in the findings. The findings suggest there are complex and overlapping factors that produce both the stigmatization experienced by CTP users that related to the ambiguous status of cannabis, lack of acknowledge about medical cannabis, and stigma associated with particular health disorders. While public acceptance of cannabis continues to grow, it appears that CTP users remain highly vulnerable to stigma at both interpersonal and institutional levels. Participant experiences of stigma related to CTP use stemmed from external sources, including their friends, family, healthcare providers, and law enforcement, and from their own internalized guilt and discomfort related to using a medication that is also often used recreationally and illegally. In addition, victim blaming discourse was evident, whereby the illness for which CTP was used attracted harsh judgements about the person’s previous health practices (e.g., HIV/AIDS in homosexual and IV drug users, smokers who get cancer) and the validity of their treatment requests. Suspicion about previous risky behaviours was prompted by CTP use and interpreted as emerging from irresponsible acts and disregard for self-health. In addition, illnesses for which others adjust or adequately cope with using conventional medical treatments, rendered suspect the use of CTP as a legitimate course of treatment.

Stigmatization related to cannabis as a substance and its illegal status are clearly intertwined. Historically, cannabis was made illegal not because of problems associated with its use, but rather, as a result of propaganda that encouraged the public to view cannabis as risky and untoward in order to reify its criminal classification [37]. Engaging in illegal activities, more generally, is stigmatized in society. Criminalizing activities render them deviant, and it is generally assumed within society that there is a good reason for this status. Even though deviance and criminality were not central to the majority of participants’ self concepts, “disidentifiers” [10] were commonly used to distance themselves from these labels. For these individuals who were already living with a chronic, often life-limiting illness and on the margins of society, this additional form of stigmatization increased the physical and emotional distress they experienced.

Even more problematic from a human rights perspective is the potential for discrimination in the healthcare system, where individuals fail to receive appropriate assessment and treatment for a health condition because of being labeled as drug dependent or a pothead. In this context, patient-provider consultations become focused on extraneous issues, such as addiction and one’s moral fiber, rather than the larger concerns of symptom management and the underlying pathology of illness. Amid this preoccupation resides an uneasiness and lingering doubt that CTP use is contrived and manipulative, whereby cannabis is masking, and in many cases adding to, the individual’s and societal problems. This discourse threatens the trust essential for a caring patient-provider relationship and may disrupt future care-seeking behaviour by patients as well as the delivery of efficacious treatments by healthcare providers. Physicians, in particular, have the obligation and duty to provide safe, competent, and ethical care to all individuals in accordance with current and accepted standards of practice [38]. Although CTP remains in the hinterland of accepted standards of practice within North America, the growing body of evidence supporting its use as a medical treatment and its availability through an established federal health program is forcing the hand of physicians and other healthcare providers to consider the potential value of cannabis as a therapeutic agent. To not do so could be potentially viewed as a breach in care and a discriminatory action.

The Supreme Court of Canada recognized that it is constitutionally problematic to put people in a position to have to choose between their liberty and their health, and this led to the establishment of the federal medical cannabis programme [39,40]. And while there continue to be advancements in the rights of CTP users at the judicial level, they are often on a case by case basis, and incidents of discrimination continue to be documented and arrests are common [41,42]. All participants in this study were either MMAD licence holders or medical cannabis dispensary members, meaning that their use of CTP was legitimate (i.e., it was for a documented medical condition). However, only those with MMAD licences who procured CTP from Canada’s contracted producer were using CTP legally. For some, choosing the legal government route was a way to quell their internal concerns about acting lawfully. However, it was apparent from our interviews that this did not necessarily relieve external stigma. Outing themselves as CTP users made them feel more vulnerable, and some actually found themselves facing more external stigma than if they had been hiding their use. It appears that due to the overarching illegal status of cannabis outside of the narrow exception for therapeutic use, the legal route does not necessarily alleviate stigma for CTP users.

Although the use of CTP appeared to be a marker of individual expression or identity, not unlike some recreational users experiencing stigma, fear of shame and loss of status necessitated efforts to manage stigma. Management of personal information and others’ knowledge of CTP use appear to be of critical importance to CTP users, with many choosing between hiding their use from others in order to pass as normal to avoid sanctions (i.e., *social avoidance)* or being open about it (*selective or indiscriminate disclosure*) in an attempt to inform others about CTP and assist with redefining users as “normal” law-abiding citizens [43]. These reactions are common in the stigma literature and both serve as an attempt to protect oneself from further stigma [44,45]. Study participants’ efforts to be responsible and discrete in their CTP use to avoid drawing attention (particularly from law enforcement) are similar to those observed among both therapeutic and recreational cannabis users [3,23,46]. The fact that some participants chose to be open about their CTP use may reflect established coping strategies developed in response to long-standing stigmatizing illnesses.

While many study participants took it upon themselves to educate others about the value of cannabis as a medicine, it is unrealistic that the work of stigma reduction rest solely on individuals compromised by health problems. Instead, formal education programs and policy reform is required that targets healthcare providers, law enforcement personnel, government authorities, as well as members of general society. Interventions that address the history of cannabis criminalization, as well as the legitimacy of CTP use and the options for legal CTP use, would go a long way to ensuring CTP users experience the full spirit of their constitutional right to health without fearing legal repercussions or experiencing the stigma of being associated with an illegal activity. Such programs could be modelled after other successful stigma reduction interventions that have been developed for other marginalized groups, including HIV/AIDS and mental illness [47-49].

Several limitations to this research are recognized. Participants were from British Columbia, a Canadian province known for its illegal cannabis production and tolerance of recreational use. The contradictions experienced by the CTP users in this study cannot be understood apart from the social and structural conditions that influenced how users viewed themselves and how they are viewed by others. Experiences of and reactions to using CTP may have differed if participants had been recruited from more conservative regions. As most of the participants indicated they were long-term users and had made the decision to use CTP several years before, their experiences of stigma may not be the same as those who have just begun to use CTP. Furthermore, the participants were self-selected (i.e., they were willing to speak openly about CTP). As a result, it could be that those who had experienced more negative stigma while using CTP, those who no longer used CTP for fear of its social and legal ramifications or who did not want to be a magnet for their friends’ or families’ discontent were thus likely underrepresented in this study. Further research is required to examine how experiences of stigma evolve over the course of CTP treatment and among different populations in different legal/social climates.

## Conclusion

Experiences of stigma among those with illness and the role stigma plays in seeking treatment are not new in the literature. However, in this literature it is not necessarily the treatment that is stigmatized, but the illness for which the treatment is used. CTP stands as one of the few treatments where users are directly stigmatized for their use of it regardless of their particular illness. The findings of this study shed light on how individuals using CTP experience stigma, and the effect on their physical and emotional wellbeing as well as the impact on healthcare interactions. The stigmatization of CTP users is related to the ambiguous status of cannabis (an illegal substance and a legal therapeutic agent at the same time), and to the lack of acknowledge about medical cannabis among the public, physicians, and law enforcement personnel. The findings reinforce the urgent need for finding better solutions and strategies to reduce stigmatization associated with use of CTP.

## Competing interests

There are no competing interests to report.

## Authors’ contributions

JLB and LGB were the principal study investigators, contributed to the conceptualization, design, conduct and analyses of the study, interpretation, and writing of this manuscript. LJLB, JLO, NRC, JB contributed to the conceptualization, design, analysis and interpretation of the data, and writing of the manuscript. All authors read and approved the final manuscript.

## References

[B1] Tramer MR Carroll D Campbell F Reynolds DJM Moore A McQuay H Cannabinoids for control of chemotherapy induced nausea and vomiting: Quantitative systematic review BMJ 2001 323 16 1 8 11440936 10.1136/bmj.323.7303.16 PMC34325

[B2] Ware M Rueda S Singer J Kilby D Cannabis use by persons living with HIV/AIDS: Patterns and prevalence of use J Cannabis Therapeutics 2003 3 2 3 15 10.1300/J175v03n02_02

[B3] Hathaway AD Cannabis users' informal rules for managing stigma and risk Deviant Behav 2004 25 6 559 577 10.1080/01639620490484095

[B4] Tjepkema M Use of cannabis and other illicit drugs Health Rep 2004 15 4 41 47 15346727

[B5] Stockwell T Sturge J Jones W Fischer B Carter C Cannabis use in British Columbia: Patterns of use, perceptions, and public opinion as assessed in the 2004 Canadian Addiction Survey - Bulletin 2 2004 Centre for Addictions Research of British Columbia (CARBC), Vancouver, Canada http://carbc.ca/Portals/0/PropertyAgent/558/Files/19/CARBCBulletin2.pdf

[B6] Savas D Public opinion and illicit drugs: Canadian attitudes towards decriminalizing the use of marijuana 2001 The Fraser Institute, Vancouver, Canada

[B7] Hathaway AD Erickson PG Lucas P Canadian public opinion on cannabis: How far out of step is it with the existing law? Can Rev Soc Pol 2007 59 44 55

[B8] Johnson BD Ream GL Dunlap E Sifaneck SJ Civic norms and etiquettes regarding marijuana use in public settings in New York City Subst Use Misuse 2008 43 7 895 918 10.1080/10826080701801477 18570024 PMC2562729

[B9] Lucas P Moral regulation and the presumption of guilt in Health Canada's medical cannabis policy and practice Int J Drug Policy 2009 20 4 296 303 10.1016/j.drugpo.2008.09.007 19124233

[B10] Goffman E Stigma: Notes on the management of spoiled identity 1963 Prentice - Hall, Englewood Cliffs, NJ

[B11] Link B Struening E Rahav M Phelan J Nuttbrock L On stigma and its consequences: Evidence from a longitudinal study of men with dual diagnoses of mental illness and substance abuse J Health Soc Behav 1997 38 2 177 190 10.2307/2955424 9212538

[B12] Weiss MG Ramakrishna J Somma D Health-related stigma: Rethinking concepts and interventions Psychol Health Med 2006 11 3 277 287 10.1080/13548500600595053 17130065

[B13] Young M Stuber J Ahern J Galea S Interpersonal discrimination and the health of illicit drug users Am J Drug Alcohol Abuse 2005 31 3 371 391 10.1081/ADA-200056772 16161724

[B14] Corrigan PW The impact of stigma on severe mental illness Cogn Behav Pract 1998 5 2 201 222 10.1016/S1077-7229(98)80006-0

[B15] Holzemer WL Human S Arudo J Rosa ME Hamilton MJ Corless I Exploring HIV stigma and quality of life for persons living with HIV infection J Assoc Nurses AIDS Care 2009 20 3 41 50 10.1016/j.jana.2009.02.002 19427593

[B16] Jacoby A Snape D Baker A Epilepsy and social identity: The stigma of a chronic neurological disorder Lancet Neurol 2005 4 3 171 178 15721827 10.1016/S1474-4422(05)01014-8

[B17] Looper KJ Kirkmayer LJ Perceived stigma in functional somatic syndromes and comparable medical conditions J Psychosom Res 2004 57 373 378 15518673 10.1016/j.jpsychores.2004.03.005

[B18] Ahern J Stuber J Galea S Stigma, discrimination and the health of illicit drug users Drug Alcohol Depend 2007 88 2–3 188 196 17118578 10.1016/j.drugalcdep.2006.10.014

[B19] Corrigan PW Sachiko AK O'Shaughnessy J The public stigma of mental illness and drug addiction: Findings from a stratified random sample J Soc Work 2009 9 2 139 147 10.1177/1468017308101818

[B20] Krieger N Sidney S Prevalence and health implications of anti-gay discrimination: A study of black and white women and men in the CARDIA cohort Int J Health Serv 1997 27 1 157 176 10.2190/HPB8-5M2N-VK6X-0FWN 9031018

[B21] Landrine H Klonoff EA Gibbs J Manning V Lund M Physical and psychiatric correlates of gender discrimination: An application of the schedule of sexist events Psychol Women Q 1995 19 4 473 492 10.1111/j.1471-6402.1995.tb00087.x

[B22] Canadian AIDS Society Cannabis as Therapy for People Living with HIV/AIDS: ‘Our Right, Our Choice’ 2006 Canadian AIDS Society, Ottawa, Ontario

[B23] Page SA Verhoef MJ Medicinal marijuana use: Experiences of people with multiple sclerosis Can Fam Physician 2006 52 64 72 16926966 PMC1479734

[B24] Curry WNL Hyperemesis gravidarum and clinical cannabis: To eat or not to eat? J Cannabis Therapeutics 2002 2 3/4 61 81

[B25] Sandelowski M Whatever happened to qualitative description? Res Nurs Health 2000 23 4 334 340 10.1002/1098-240X(200008)23:4<334::AID-NUR9>3.0.CO;2-G 10940958

[B26] Lincoln YS Guba EG Naturalistic Inquiry 1985 Sage, Newbury Park

[B27] Sullivan-Bolyai S Bova C Harper D Developing and refining interventions in persons with health disparities: The use of qualitative description Nurs Outlook 2005 53 3 127 133 10.1016/j.outlook.2005.03.005 15988449

[B28] Canada H Information for the patient- marihuana (cannabis) 2006 Health Canada, Ottawa, ON

[B29] Canadian AIDS Society Submission of the Canadian AIDS society on the Proposed Amendments to the Marihuana Access Regulations 2004 Canadian AIDS Society, Ottawa, ON

[B30] Canadian Cancer Society Medicinal use of marijuana 2002 Canadian Cancer Society, Toronto, ON

[B31] Canadian Medical Association Journal Marijuana: Federal smoke clears, a little CMAJ 2001 164 10 1397 11387904 PMC81049

[B32] Capler R Black H Up in smoke: The illusion of legal medical marijuana Living+ 2003, Nov-Dec:19–21. https://www.thecompassionclub.org/sites/default/files/documents/BCCCS_up_in_smoke.pdf

[B33] Capler R Black H British Columbia Compassion Club Society Response to MMAR amendments 2004 British Columbia Compassion Club Society, Vancouver, BC

[B34] Mernagh Rv 2011 http://www.marijuanalaws.ca/pdf/Mernagh-Ruling-Apr13-2011.pdf

[B35] Capler R Lucas P Guidelines for the Community-Based Distribution of Medical Cannabis in Canada 2006 British Columbia Compassion Club Society and Vancouver Island Compassion Society, Vancouver, BC

[B36] Statistics Canada Low income cut-offs for 2008 and low income measures for 2007 - income research paper series 2009 Statistics Canada, Ottawa, ON

[B37] Fischer B Ala-Leppilampi K Single E Robins A Cannabis law reform in Canada: Is the "saga of promise, hesitation and retreat" coming to an end? CJCCJ 2003 45 3 265 297

[B38] The Canadian Medical Protective Association (CMPA) Medico-legal Handbook for Physicians in Canada 2010 Canadian Medical Protective Association, Ottawa, ON

[B39] Parker RV Ontario Court of Appeal, ONCA 5762 2000 http://www.ontariocourts.on.ca/decisions/2000/july/parker.htm

[B40] Lucas PG Regulating compassion: An overview of Canada's federal medical cannabis policy and practice Harm Reduct J 2008 5 5 10.1186/1477-7517-5-5 18226254 PMC2267789

[B41] Campbell C The medical use of marihuana and the right to equality 2009 Research and Planning Branch of the Commission des Droits de la Personne et des Droits de la Jeunesse, Quebec, Canada

[B42] Patriquin M The limits on compassion Macleans 2010 [Online]. http://www2.macleans.ca/2010/07/01/the-limits-on-compassion/ [2010, 07/25]

[B43] Corrigan PW Matthews AK Stigma and disclosure: Implications for coming out of the closet J Ment Health 2003 12 3 235 248 10.1080/0963823031000118221

[B44] Sirey JA Bruce Ml Alexopolous GS Perlick DA Raue P Friedman SJ Perceived stigma as a predictor of treatment discontinuation in young and older outpatients with depression Am J Psychiatry 2001 158 479 481 10.1176/appi.ajp.158.3.479 11229992

[B45] Lichtenstein B Stigma as a barrier to sexually transmitted infection in the American deep south: Issues of race, gender and poverty Soc Sci Med 2003 57 2435 2445 10.1016/j.socscimed.2003.08.002 14572849

[B46] Hathaway AD Comeau NC Erickson PG Cannabis normalization and stigma: Contemporary practices of moral regulation Criminol Crim Justice 2011 11 5 451 469 10.1177/1748895811415345

[B47] Brown L Macintyre K Trujillo L Interventions to reduce HIV/AIDS stigma: What have we learned? AIDS Educ Prev 2003 15 1 49 69 10.1521/aeap.15.1.49.23844 12627743

[B48] Heijnders M Van der Meij S The fight against stigma: An overview of stigma-reduction strategies and interventions Psychol Health Med 2006 11 3 353 363 10.1080/13548500600595327 17130071

[B49] Gaebel W Baumann AE Interventions to reduce the stigma associated with severe mental illness: Experiences from the open doors program in Germany Can J Psychiatry 2003 48 10 657 662 14674047 10.1177/070674370304801003

